# Reversible global hypoperfusion in an adult patient with a mixed diabetic ketoacidosis/hyperglycemic hyperosmolar coma: A case report

**DOI:** 10.1002/ccr3.5576

**Published:** 2022-04-04

**Authors:** Valentina Dafni Petroulia, Christoph Kurmann, Matthias Haenggi, Franca Wagner, Arsany Hakim

**Affiliations:** ^1^ University Institute of Diagnostic and Interventional Neuroradiology, Inselspital Bern University Hospital, and University of Bern Bern Switzerland; ^2^ Department of Intensive Care Medicine, Inselspital Bern University Hospital, and University of Bern Bern Switzerland

**Keywords:** diabetes mellitus, diabetic ketoacidosis, global hypoperfusion, neuroimaging

## Abstract

Diabetic ketoacidosis is a severe complication of diabetes mellitus. We report a case of global hypoperfusion in an elderly patient on CT, with complete resolution shown on early MRI follow‐up. Metabolic causes have always to be included in the differential diagnosis of diffuse hypoperfusion in the appropriate clinical setting.

## Graphical abstract

Diabetic ketoacidosis is a severe complication of diabetes mellitus. We report a case of global hypoperfusion in an elderly patient on CT with complete resolution on early follow‐up imaging. Metabolic causes have always to be included in the differential diagnosis of diffuse hypoperfusion in the appropriate clinical setting.

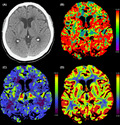

## 1 INTRODUCTION

Diabetic ketoacidosis (DKA) is a serious complication of insulin‐dependent diabetes mellitus associated with high mortality in both children and adults. It is primarily observed in patients with type 1 and less often in patients with type 2 diabetes. The pathophysiology of DKA and hyperglycemic hyperosmolar syndrome is different, but both are the result of insulin deficiency leading to an increased concentration of the counterregulatory hormones.^1^ Treatment with insulin, rehydration, and monitored serum electrolyte correction targets the hyperglycemia and hyperosomolarity as well as the electrolyte disturbances.

Few studies have examined the neuroimaging findings in patients with DKA and hyperglycemic hyperosmolar coma, and most of the case reports have focused on children and adolescents.^2^, ^3^, ^4^ In adult patients with type 1 or 2 diabetes, cerebral edema seems to be the most common finding, followed by stroke with or without hemorrhagic transformation.^1^, ^4^

In this case report, we discuss a patient with DKA and hyperosmolar hyperglycemic coma with diffuse hypoperfusion visible on computed tomography (CT) perfusion imaging. To the best of our knowledge, this is the first human perfusion study demonstrating global hypoperfusion before or at initiation of treatment for DKA.^5^ No alterations of brain parenchyma were seen on the CT scan or the follow‐up MRI scan after 5 h.

## 2 CASE PRESENTATION

A 77‐year‐old woman with a history of diabetes mellitus type 2 treated with the dipeptidyl peptidase‐4 (DPP‐4)‐inhibitor sitagliptin presented to our emergency department with a loss of consciousness (Glasgow coma scale of 10, E3/V2/M4). She was found unconscious in her apartment with an unknown time last‐seen‐well. Her forehead showed old signs of injury from a fall. Upon admission, the patient presented with tachypnea, tachycardia, hypothermia, and hypotonia. Her pupils were dilated, with sluggish response. Blood samples were drawn, and while awaiting the results, CT, CT angiography, and CT perfusion were requested to rule out neurological causes of coma, such as stroke, seizure (“non‐convulsive status epilepticus”), hemorrhage, or brain tumor.

Laboratory tests indicated hyperglycemia with initial serum glucose of 44 mmol/L (normal range (NR): 4.56–6.38 mmol/L) and glycated hemoglobin (HbA1c) of 15.9% (NR: 4.8%–5.9%). High concentrations of serum and urine ketones were found as well as high osmolality: 368 mOsm/kg (NR: 280–295 mOsm/kg), osmol gap 18 mOsm/kg (NR: <10 mOsm/kg), anion gap 39 mmol/L (NR: 3–11 mmol/L), 3‐ beta‐hydroxybutyrate 11395 µmol/L (NR: <150 µmol/L). Arterial blood pH was 6.8 (NR: 7.35–7.45), pCO_2_ 17 mmHg (NR: 32–43 mmHg), base excess −27.1 mmol/L (NR: −2.5 to +2.3 mmol/L), lactate 3.4 mmol/L (NR: 0.63–2.44 mmol/L), serum urea 11.9 mmol/L (NR: 3.5–7.2 mmol/L), and C‐reactive protein 11 mg/L (NR: <5mg/L). The results of laboratory tests were consistent with severe ketoacidosis and mild lactic acidosis as well as acute kidney injury.

**FIGURE 1:**
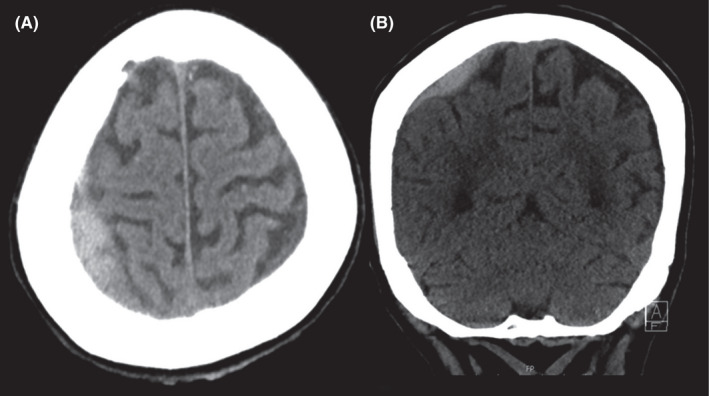
In the non‐enhanced CT scan, a right‐sided acute small (max. 8 mm) subdural hematoma, and a left‐sided chronic small (max. 3 mm) subdural hematoma were seen on the axial (A) and coronal (B) CT slices

**FIGURE 2:**
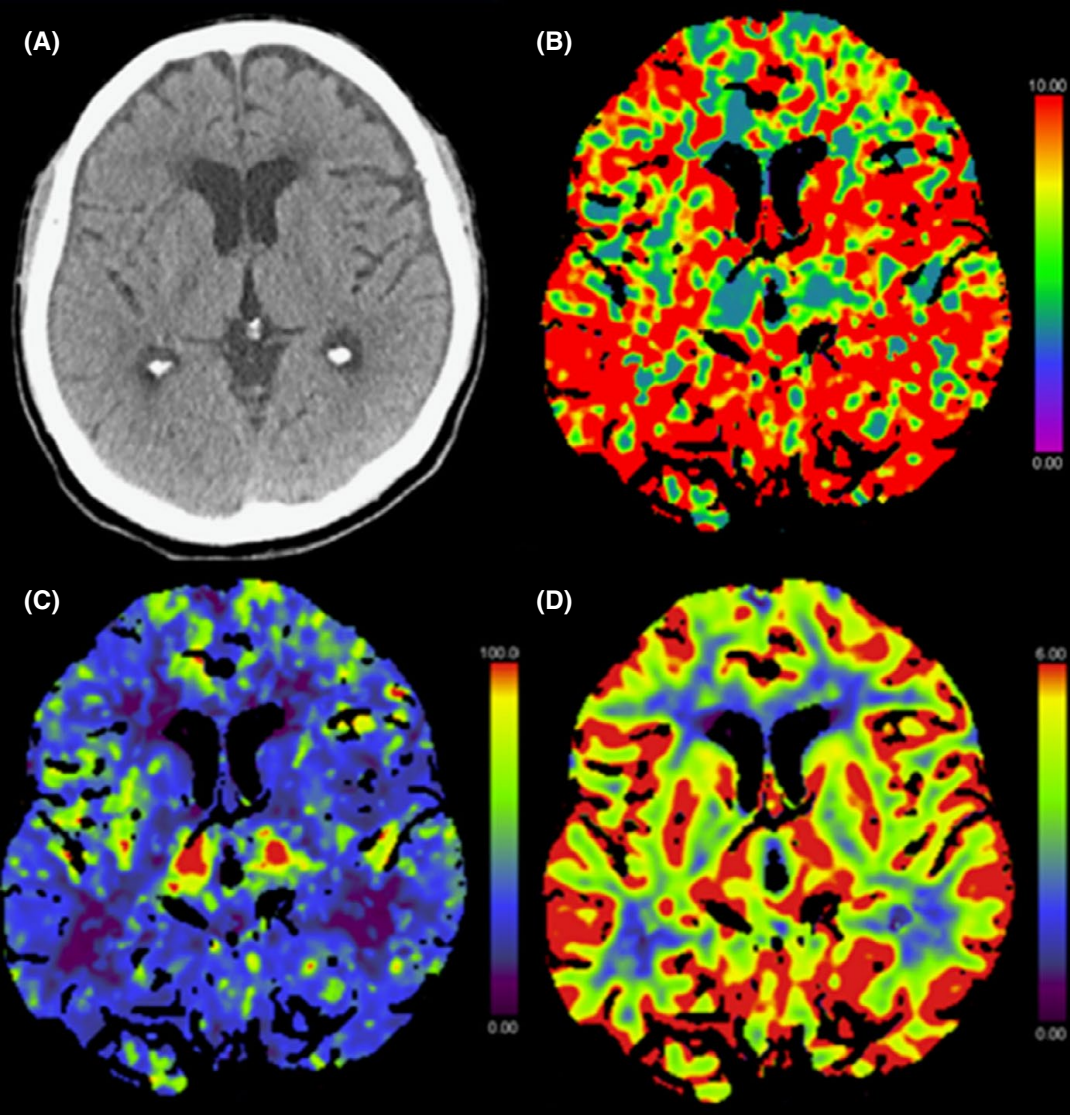
Initial axial non‐enhanced computed tomography (CT) (A) showing normal parenchyma with no evidence of swelling or ischemic changes. CT perfusion showing prolonged mean transit time (MTT) (B) with global cortical hypoperfusion including the basal ganglia in the cerebral blood flow map (C), but no abnormalities in the cerebral blood volume map (D)

Shortly before the acquisition of the CT scan, treatment with insulin and fluid substitution as well as bicarbonate and thiamine was initiated. There was no clinical improvement, so the CT scan was not canceled. In the non‐enhanced CT scan, a right‐sided acute small (max. 8 mm) subdural hematoma and a left‐sided chronic small (max. 3 mm) subdural hematoma were seen (Figure 1). No edema or infarction of the brain parenchyma was present (Figure 2). The extra‐ and intracranial arteries showed no pathological findings. Post‐contrast CT showed no abnormal enhancement of the brain parenchyma or cerebral venous thrombosis.

The perfusion study revealed a global diffuse reduction in the cerebral blood flow (CBF), sparing the thalami and brainstem, with delay in all temporal parameters (mean transit time (MTT), time to maximum (T*_max_*), time to peak (TTP), and time to drain (TTD)), but no pathologies in the cerebral blood volume (CBV), (Figure 2). There was no territorial ischemic pattern. Quantitative analysis of the perfusion showed a significant CBF reduction in the gray matter to 24 ml/100g/min (NR: 60.3–69.7 ml/100g/min) and associated prolongation of the temporal parameters in gray and white matter, confirming the findings of the qualitative analysis (Table 1).

**TABLE 1:** Values of the quantitative analysis of computed tomography (CT) perfusion with regions of interest placed in the cortex (gray matter) and centrum semiovale (white matter)

	Cortical	Normal range gray matter	Centrum semiovale	Normal range white matter
CBF	24	60.3–69.7 ml/100g/min	16	27.3–32.7 ml/100g/min
CBV	4.8	3.3–3.7 ml/100g	2	1.7–2.1 ml/100g
MTT	11.9	3–3.6 s	8.6	3.4–4.6 s
TTP	17.9	5.7–10.5 s	16.1	6.8–11.8 s
TTD	13.6	3.1–4.3 s	10.8	3.8–5.8 s

Abbreviations: CBF, cerebral blood flow; CBV, cerebral blood volume; MTT, mean transit time; TTD, time to drain; TTP, time to peak.

Because of the global pattern of hypoperfusion of brain parenchyma, hypoxic‐ischemic brain injury was considered as the main differential diagnosis.

After the CT scan, the patient was intubated because of respiratory depression and transferred to the intensive care unit.

**FIGURE 3:**
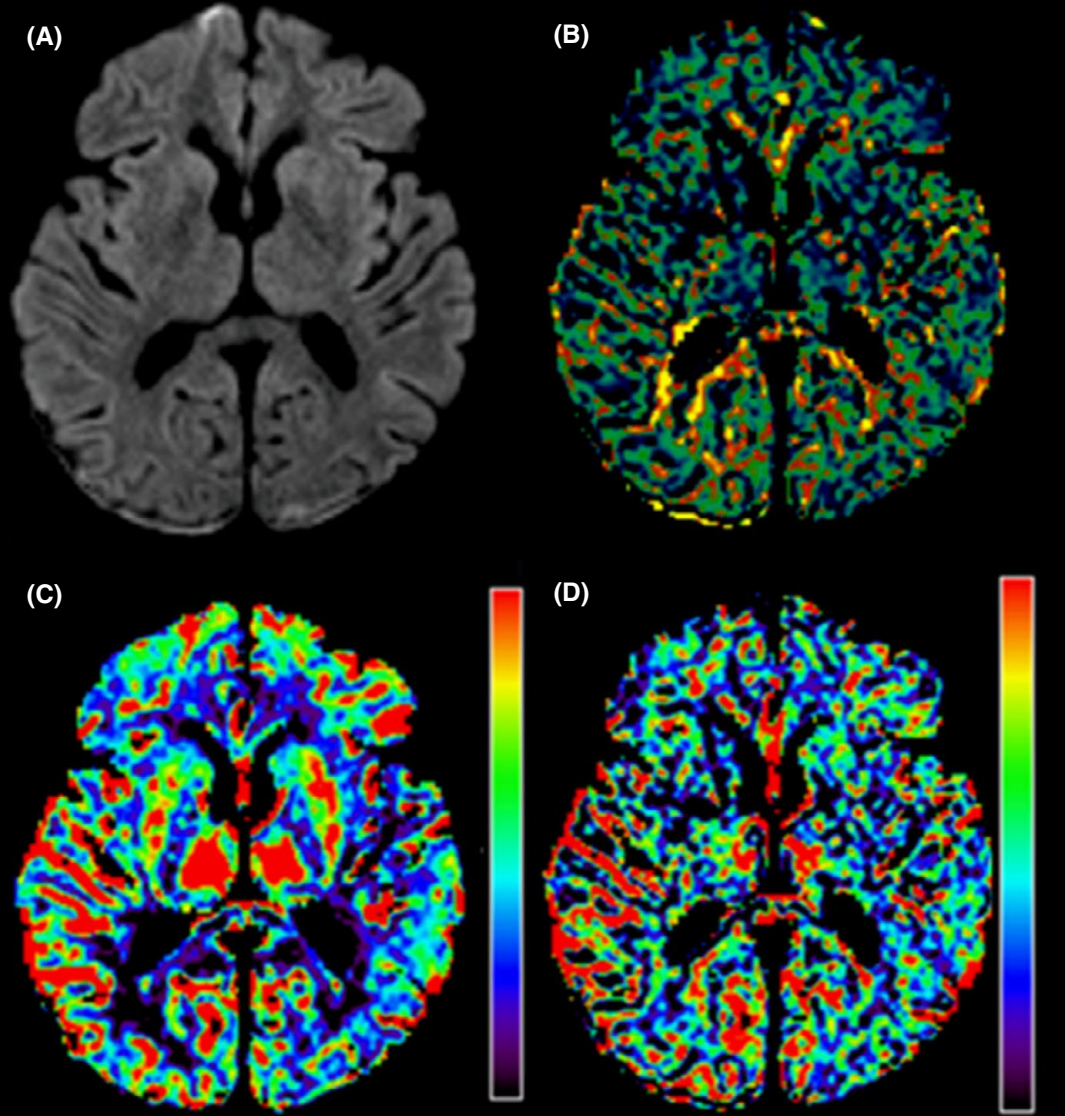
Follow‐up magnetic resonance imaging 5 hours after the initial computed tomography exam showing no diffusion restriction in the diffusion‐weighted imaging sequence (A). Note the bilateral occipital subdural hematoma. The MR perfusion shows normalization of the perfusion parameters with reversible changes in mean transit time (MTT) (B) and cerebral blood flow maps (C). No changes are apparent in the cerebral blood volume map (D)

MRI of the brain was performed 5 h after the CT scan to investigate the possibility of ongoing cerebral edema and to follow up on the cerebral perfusion of the patient. Blood glucose had decreased to 29 mmol/L, pH was 7.24, pCO_2_ 17 mmHg, base excess −19.5 mmol/L, and lactate 2.8 mmol/L. Diffusion‐weighted imaging showed no cytotoxic edema, that is, no diffusion restriction. Like the CT examination, MRI showed no effacement of the sulci or narrowing of the ventricles, and the width of the cerebrospinal fluid (CSF) spaces was unchanged. The T2‐weighted and fluid‐attenuated inversion recovery (FLAIR) sequence showed no cerebral edema and no other pathological findings related to hyperglycemia or electrolyte disturbances. Small bilateral parietal and occipital subdural collections were noted, in line with the CT findings. There was no occlusion of the intracranial arteries. The superficial and deep venous system of the brain was patent. Susceptibility‐weighted imaging showed no parenchymal microbleeds. Similarly, no parenchymal or leptomeningeal contrast enhancement was found. MR perfusion showed normal perfusion patterns with normalization of the hypoperfused regions seen on the previous CT perfusion study (Figure 3).

The patient remained in the intensive care unit for the next two days. After extubation, stabilization of her clinical condition, and further laboratory test results, she was transferred to a medical ward. Following normal laboratory test results, negative blood and urine cultures, the patient was discharged after seven days and transferred to a nursing facility. In the long term, the patient was able to return home in a good clinical condition. According to the family, mild cognitive decline has been noted for years, but accelerated 3 months before the described episode. This DKA episode was attributed to mild cognitive impairment and the fact that the patient has probably forgotten to take her prescribed insulin. A tendency for falls has also been noted before this episode, which explains the subdural hematoma. One year after the episode, the patient is still living at home alone, but with the help of the family and daily professional nursing support. No further hospital admissions were necessary, and an appointment in the memory clinic was scheduled 11 months after the hospitalization, indicating a further and steady cognitive decline.

## 3 DISCUSSION

DKA is a serious clinical complication, occurring mostly in children and adolescents newly diagnosed with diabetes mellitus type 1, and is associated with high morbidity and mortality. An extremely serious complication of DKA is cerebral edema, which is often associated with the development of severe neurological deficits. Adults with DKA have higher mortality than children.^4^

Neuroimaging has become a key diagnostic tool in the emergency department, especially in patients with unclear loss of consciousness. CT and MR imaging are able to promptly rule out intracranial pathologies in the emergency setting and to initiate the correct therapeutic procedure and medication depending on the (neuro‐)radiological findings. Especially in a comatose patient, life‐saving management could be initiated while waiting for the blood test results; therefore, it is very important to exclude intracranial pathologies that require immediate action like a large intracranial hematoma that requires evacuation or a brain infarction due to a large vessel occlusion that requires mechanical thrombectomy. In our patient, the neuroimaging did not influence the therapeutic procedure but excluded other relevant brain pathologies that clinicians have to be aware of in a comatose emergency patient.

The first scan of our patient showed diffuse cerebral hypoperfusion sparing the thalami and brainstem. This is surprising, because acidosis usually increases brain perfusion; acidosis is the main mechanism for matching regional CBF with demand. The diffuse cerebral hypoperfusion and reduced CBF could be related to the patient's metabolic acidemia or to the osmolar disturbances caused by the hyperglycemia as well as due to neuroinflammatory response caused by DKA.^6^ Another possible mechanism is an intracellular energetic failure due to deficiency of insulin, which is required to transport glucose into the cells, leading to a state of diminished energy in a similar way to hypoxia,^5^ but pure hyperketonemia without acidosis would induce an increase in CBF, which was not seen in our case.^7^

Hyperglycemia may also cause endothelial dysfunction that affects regional cerebral perfusion and could, therefore, explain the increased ischemic risk in patients with uncontrolled diabetes.^8^ However, no ischemic lesion or cerebral edema was detected in our patient either in the initial CT scan or in the follow‐up MRI, most probably due to rapid initiation of treatment in combination with adequate compensatory mechanism of the cerebral vasculature (i.e., intact autoregulation).^6^

Up to now, the imaging findings in children and adults with DKA in the acute setting have only been published as case reports, mostly describing single cases of cerebral edema, cerebral venous thrombosis, posterior reversible encephalopathy syndrome, diabetic encephalopathy, or secondary metabolic encephalopathy.^2^, ^4^, ^9^

Studies on animal models have provided evidence of CBF reduction in animals with DKA, but to our knowledge, only one study in adults using single‐photon emission computed tomography (SPECT) has shown changes in regional cerebral perfusion associated with fasting plasma glucose and increased CBF after normalization of glycemia.^8^, ^10^ Global hypoperfusion may be caused by several conditions, such as diffuse global cerebral edema, brain death, and hypoxic‐ischemic injury. All of these were ruled out in our case. Important pitfalls to be aware of in assessing patients with global hypoperfusion are cardiac or technical factors such as low cardiac output, low injection rate of contrast agent, or poor placement of regions of interest during the postprocessing of the perfusion study, all of which were ruled out in our case.

Luckily, the brain hypoperfusion did not result in a permanent deficit, which can be expected in prolonged DKA,^2^, ^11^, ^12^ and attributed to the reduction in blood flow during DKA. We hypothese that the early initiation of treatment reverted the documented perfusion deficit and lead to the favorable outcome.

## 4 CONCLUSION

Our case report describes reversible diffuse cerebral hypoperfusion of gray and white matter in a patient with uncontrolled diabetes mellitus and DKA‐induced coma with associated hyperglycemia without accompanying cerebral edema. Hence, metabolic causes such as DKA should be included in the differential diagnosis of diffuse hypoperfusion in the appropriate clinical setting.

## AUTHOR CONTRIBUTIONS

Valentina Dafni Petroulia designed the case report, analyzed the imaging data, and drafted the manuscript. Christoph Kurmann obtained and analyzed the imaging data and drafted the manuscript. Matthias Haenggi obtained and analyzed the clinical data and drafted and revised the manuscript. Franca Wagner drafted and critical revised the manuscript and contributed to overall supervision. Arsany Hakim contributed to supervision of the analyses of imaging data, drafted, and critical revised the manuscript.

## CONSENT

Written consent has been obtained from the patient and the patient's daughter for publication of the case report and sharing of the images in a de‐identified manner.

## Data Availability

The data that support the findings of this study are available on request from the corresponding author. The data are not publicly available due to privacy or ethical restrictions.
